# The deubiquitinase OTUB1 fosters papillary thyroid carcinoma growth through EYA1 stabilization

**DOI:** 10.1111/jcmm.17020

**Published:** 2021-11-12

**Authors:** Peiyi Xie, Qing Chao, Jiuang Mao, Yue Liu, Jiayu Fang, Jing Xie, Jing Zhen, Yongqi Ding, Bidong Fu, Yun Ke, Da Huang

**Affiliations:** ^1^ Department of General Surgery Second Affiliated Hospital of Nanchang University Nanchang China; ^2^ Second College of Clinical Medicine Zunyi Medical University Zhuhai China; ^3^ Department of Oncology the First Affiliated Hospital of Soochow University Suzhou China; ^4^ Second College of Clinical Medicine Nanchang University Nanchang China; ^5^ Department of Thyroid Surgery Second Affiliated Hospital of Nanchang University Nanchang China

**Keywords:** deubiquitylation, EYA1, OTUB1, papillary thyroid cancer, proliferation

## Abstract

Deubiquitinating enzyme OTU domain‐containing ubiquitin aldehyde‐binding proteins 1 (OTUB1) has been shown to have an essential role in multiple carcinomas. However, the function of OTUB1 in papillary thyroid cancer (PTC) and the underlying mechanisms regulating PTC cells proliferation remain poorly understood. In this study, OTUB1 was significantly upregulated in papillary thyroid carcinoma tissues and cells. Through *in vitro* and *in vivo* experiments, knockdown of OTUB1 suppressed PTC cells growth whereas OTUB1 overexpression enhanced the proliferation ability of PTC cells. Moreover, the eyes absent homologue 1 (EYA1) was recognized as a potential target of OTUB1 through mass spectrometry analysis, and we further verified that EYA1 protein level was positively correlated with OTUB1 expression in PTC cells and clinical samples. Mechanistically, OTUB1 could interact with EYA1 directly and deubiquitinate EYA1 to stabilize it. At last, EYA1 was found to play an essential role in OTUB1‐derived PTC cells growth. Overall, our investigation reveals that OTUB1 is a previously unrecognized oncogenic factor in PTC cells proliferation and suggests that OTUB1 might be a novel therapeutic target in PTC.

## 1 INTRODUCTION

Thyroid carcinoma is one of the commonest endocrine diseases worldwide. The incidence of thyroid carcinoma continues to rise in recent years.^1^ Nearly 70% to 80% of thyroid cancers belong to papillary thyroid carcinoma (PTC), and PTC is recognized as one of the commonest malignancies in endocrine system.^2^ Poor prognosis of PTC is closely correlated with clinical characteristics including tumour growth and lymph node metastasis.^3^, ^4^ Additionally, the incidence rates of PTC have rapidly increased with the use of ultrasound screening in the last few decades.^5^ Thus, it is particularly important to investigate novel therapeutic strategies for clinical therapy of papillary thyroid cancer and investigate a better understanding of the molecular mechanisms involved in the progression of papillary thyroid cancer.

Protein ubiquitination can be blocked by deubiquitylating enzymes (DUBs, also known as deubiquitinases), which contributes to cleavage of ubiquitin modifications from substrate proteins.^6^ DUBs can be subdivided into six families according to sequence and domain conservation, which include ubiquitin‐specific proteases (USPs), ubiquitin C‐terminal hydrolases (UCHs), ovarian tumour proteases (OTUs), Machado‐Josephine domain‐containing proteases, the Jab1/MPN/MOV34 metalloenzymes (JAMM, also known as MPN 1) and motif interacting with Ub‐containing novel DUB family (MINDY).^7^ Sixteen ovarian tumour (OTU) family DUBs exist in humans, and most members play important role in regulating cell‐signalling cascades.^8^ OTU domain‐containing ubiquitin aldehyde‐binding protein 1 (OTUB1) was initially identified as an OUT DUBs. Recently, OTUB1 has emerged as essential modulators of multiple carcinomas progression and accumulating evidences suggest that OTUB1 plays an essential role in negatively regulating ubiquitination to enhance stability of proteins.^9^, ^10^ In renal cell carcinoma, for example, OTUB1‐derived deubiquitylation of FOXM1 can promote cancer cells growth and metastasis.^11^ Additionally, another study also reveals that OTUB1 can influence cancer cell immunosuppression through regulating PD‐L1 stability.^12^ Importantly, aberrant upregulation of OTUB1 can enhance progression of multiple cancers including bladder cancer, prostate cancer and hepatocellular carcinoma.^13^, ^14^, ^15^ However, the function of OTUB1 in papillary thyroid cancer remains elusive.

Therefore, we investigated the clinical relevance and underlying role of OTUB1 in papillary thyroid carcinoma. Our research presented the first evidence involved in the role of OTUB1 in PTC. We observed that OTUB1 was markedly overexpressed in tissues and cells lines of PTC. Moreover, through conducting mass spectrometry analysis, we identified the oncogenic function of OTUB1 in facilitating PTC cell growth and identified EYA1 as a potential downstream target of OTUB1 in PTC cells. Furthermore, we disclosed that OTUB1 could deubiquitinate EYA1 directly to block its ubiquitination. Finally, we demonstrated that EYA1 was a critical factor in OTUB1‐mediated PTC proliferation. In summary, our research might provide a novel insight into clinical PTC treatment targeting OTUB1.

## 2 MATERIALS AND METHODS

### 2.1 Human samples collection

A total of 85 pairs PTC tissues from PTC patients undergoing surgical resection at the Second Affiliated Hospital of Nanchang University from January 2016 to March 2019 were collected. None of the patients was treated by neoadjuvant therapy. The Ethical Committee of the Second Affiliated Hospital of Nanchang University has approved the research. The expressions of OTUB1 and EYA1 were examined through Western blot and quantitative real‐time polymerase chain reaction (qRT‐PCR).

### 2.2 Cell culture and treatment

PTC cell lines KAT‐5, BCPAP, TPC‐1 and KTC‐1 were obtained from the Shanghai Institute of Cell Biology and the Type Culture Collection of Chinese Academy of Sciences. K1 cell line was obtained from the American Type Culture Collection. Human thyroid follicular epithelial cell line (Nthy‐ori 3‐1) was obtained from the European Collection of Animal Cell Cultures. Short tandem repeat analysis has confirmed authenticity of all cell lines. Dulbecco's modified Eagle's Medium (DMEM, Gibco) containing 10% foetal calf serum (FBS, Gibco) was employed to culture all cell lines. Cells were incubated in a humidified incubator containing 5% CO_2_ at 37°C.

### 2.3 Quantitative real‐time PCR

The qRT‐PCR was conducted as the previous study.^16^ The standard Trizol reagent (Invitrogen, USA) was used to isolate total RNA of cells and tissues. The PrimeScript RT Reagent Kit (Invitrogen) was used to reverse transcription, and PCR was conducted by using SYBR Premix Ex Taq (TaKaRa Bio, Japan). Primer pairs were as follows: GAPDH, forward 5′‐CCATGGGGAAGGTGAAGGTC‐3′ and reverse 5′‐TGAAGGGGTCATTGATGGCA‐3′; OTUB1, forward 5′‐ACAGAAGATCAAGGACCTCCA and reverse 5′‐CAACTCCTTGCTGTCATCCA‐3′; EYA1, forward 5′‐GTTCATCTGGGACTTGGA‐3′ and reverse 5′‐GCTTAGGTCCTGTCCGTT‐3′.

### 2.4 Western blot

Western blot was conducted according to previously described.^17^ RIPA buffer (Beyotime, China) containing protease inhibitor (Thermo Fisher Scientific, USA) was used to isolate total proteins. Then, the concentration of proteins was evaluated by BCA Protein Assay kit (Thermo Fisher Scientific). Sodium dodecylsulphonate (SDS) polyacrylamide gel electrophoresis was used to separate proteins, followed by electroblotting (Millipore, USA) which can transfer proteins onto a polyvinylidene fluoride (PVDF) membrane. Next, PVDF membranes were incubated with primary antibodies for a night at 4°C. The following antibodies were employed to examine target proteins: anti‐OTUB1 monoclonal antibody (1:500; ab175200, Abcam), anti‐EYA1 polyclonal antibody (1:500, ab194448, Abcam) and anti‐ubiquitin monoclonal antibody (1:500, sc8017, Santa Cruz). After incubation with secondary antibody (Proteintech, China) for 1 h at 37°C, protein band intensity was evaluated through Quantity One software (Bio‐Rad, USA).

### 2.5 Stable cell lines construction

The lentivirus with short hairpin RNAs targeting the OTUB1 expression was used to construct PTC cell lines with stable OTUB1 knockdown. PTC cell lines with stable OTUB1 overexpression were constructed through transfection of OTUB1‐overexpressing lentivirus vector plasmid pCDF1 (System Biosciences, China). Puromycin (Invitrogen) was used more than three weeks to select lentivirus‐infected cells.

### 2.6 EdU assay

The initial density of 1 × 10^4^ cells/well was seeded into 96‐well plates. After incubation for 20 h, cells were incubated with 5‐ethynyl‐20‐deoxyuridine (EdU; Ribobio) for another 1.5 h. Next, cells were treated by 1xApollo reaction cocktail for half an hour after being washed by PBS for 3 times. At last, the DNA contents of the cells were stained by Hoechst 33342 (5 mg/ml) for 35 min. The experimental results can be imaged by a confocal laser scanning microscope (Leica Microsystems, Germany).

### 2.7 Cell counting kit‐8 assay

Cell counting kit‐8 assay was employed to detect cell viability in indicated time. An initial concentration of 5 × 10^3^ cells was seeded into 96‐well plates. 10 μl of CCK‐8 solution (TaKaRa, Japan) was added to each well following the manufacturer's instructions. The absorbance at wavelength of 450 nm was obtained after 1h incubation.

### 2.8 Co‐immunoprecipitation experiment

Cell lysis was incubated with IgG and protein A+G Agarose (Thermo Fisher Scientific) for 2 h at 4°C to eradicate unspecific binding. Next, indicated primary antibody was added at 4°C overnight. The protein A/G‐agarose was then separated through centrifugation. After heating for 20 min at 100°C with loading buffer added, the immunoprecipitated proteins were detected through gel electrophoresis and immunoblotting analysis. At last, Quantity One software (Bio‐Rad) was employed to analyse protein bands intensity.

### 2.9 Xenografts mouse model

PTC cells (2 × 10^6^) with OTUB1 stably knockdown or overexpression were inoculated into the flanks of 4‐week‐old female nude mice (Model Animal Research Center, China). Caliper was used to evaluate sizes of tumours every five days. The calculation formula is *V* = [length/2] × [width^2^].^18^ On the 30th day, all mice were sacrificed and evaluated for tumour weights. All animal procedures were permitted by the Ethics Committee for Animal Experiments of the Second Affiliated Hospital of Nanchang University.

### 2.10 Statistical analysis

Statistical analyses were carried out by GraphPad Prism (version 5), and *p* < 0.05 was considered significant. Statistical analyses between two groups were analysed by Student's *t* test, and multiple comparisons were analysed by ANOVA. Data were expressed as mean ± SD from at least three independent experiments.

## 3 RESULTS

### 3.1 OTUB1 is aberrantly overexpressed in PTC tissues and cell lines

**FIGURE 1:**
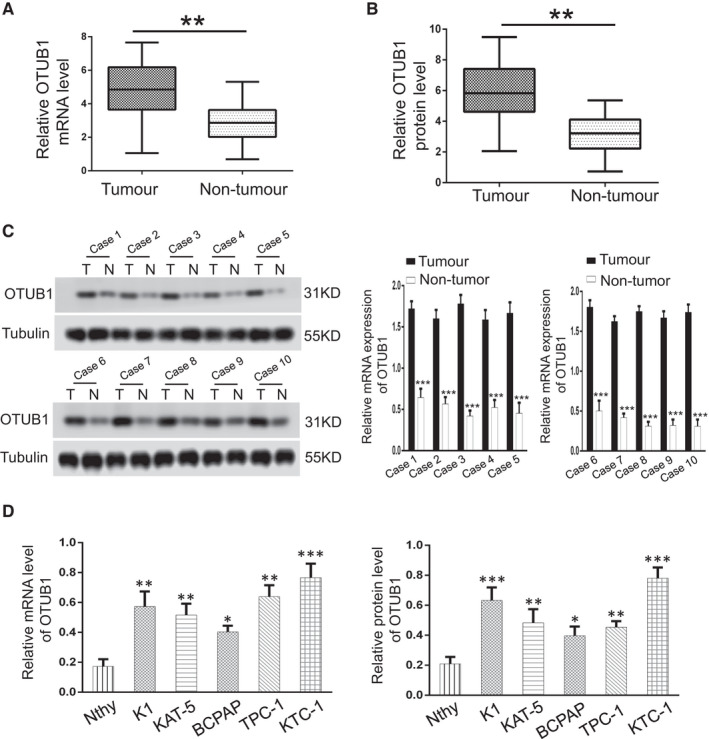
OTUB1 expression is highly upregulated in human PTC. (A) qRT‐PCR analysis of OTUB1 mRNA expression in 85 cases of PTC tissues and adjacent noncancerous samples. (B) Relative protein level of OTUB1 in 85 pairs PTC tissues and adjacent normal tissues through Western blot. (C) Representative images and quantitative analysis of OTUB1 expression in 10 cases PTC samples and noncancerous adjacent tissues through Western blot and qRT‐PCR analysis. (D) OTUB1 mRNA and protein expression analysis in normal thyroid cell and PTC cell lines. *p < 0.05, **p < 0.01

To explore the underlying function of OTUB1 in PTC proliferation, we firstly examine the expression of OTUB1 in 85 cases of human PTC samples through qRT‐PCR and Western blot. The qRT‐PCR data revealed that the mRNA expression of OTUB1 was increased in 85 PTC samples in comparison with adjacent normal tissues (Figure 1A). Additionally, Western blot also demonstrated that OTUB1 protein level was dramatically upregulated compared with noncancerous adjacent tissues (Figure 1B). Then, we selected 10 cases of 85 pairs PTC specimens and it was observed that the protein and mRNA level of OTUB1 was highly increased in PTC tissues in comparison with nontumourous adjacent tissues (Figure 1C). Furthermore, we examined the mRNA and protein level of OTUB1 in normal thyroid cells and multiple PTC cell lines. As expected, we observed that OTUB1 expression was markedly increased in PTC cell lines in comparison with normal thyroid cell (Figure 1D). Collectively, these data reveal that OTUB1 is aberrantly overexpressed in PTC tissues and cell lines.

### 3.2 OTUB1 knockdown inhibits PTC cell proliferation *in vitro* and *in vivo*

**FIGURE 2:**
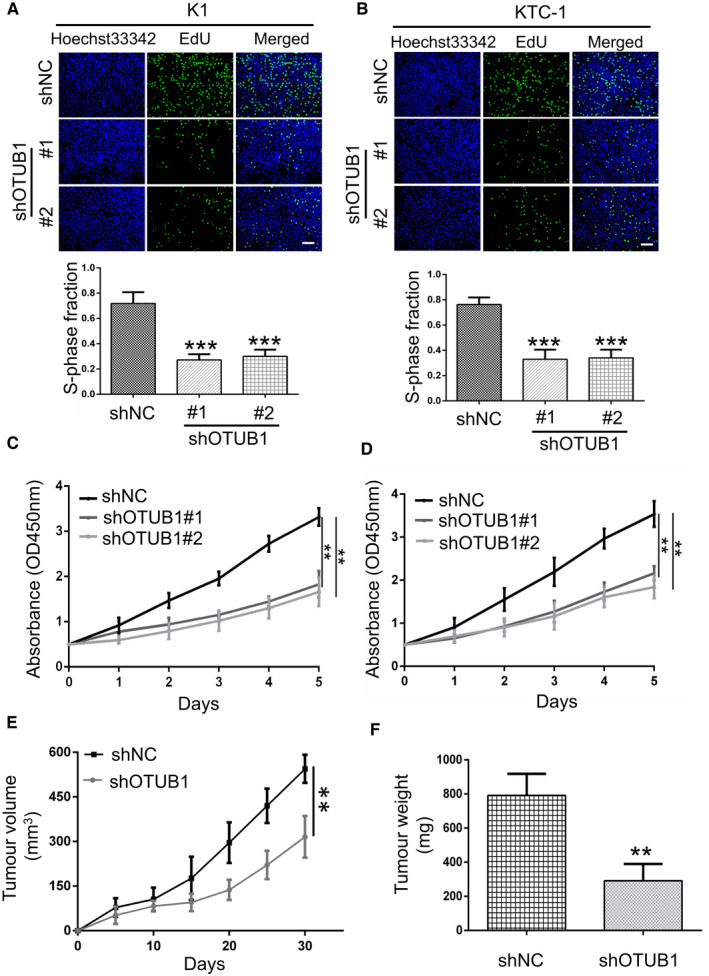
OTUB1 knockdown suppresses PTC cell growth *in vitro* and *in vivo*. (A and B) EdU assay showing the proliferation capability for K1 and KTC‐1 cells transfected with control shRNA or shOTUB1 RNA. (C and D) CCK‐8 assay was used to determine the growth capability of K1 and KTC‐1 cells with OTUB1 downregulation or not. (E and F) Tumour size and tumour weight of OTUB1‐shNC or OTUB1‐shOTUB1 group of nude mice were measured and correlated tumour growth curves were completed. *p < 0.05, **p < 0.01, scale bar, 100 μm

To investigate the function of OTUB1 in fostering PTC cells proliferation, we employed lentiviral vectors harbouring shRNA sequence of OTUB1 to suppress the expression of OTUB1 in PTC cells. Through performing EdU assay and CCK‐8 assay, we detected the growth ability of K1 and KTC‐1 cells with OTUB1 knockdown compared with the control group. Our results demonstrated that OTUB1 suppression dramatically inhibited the growth capability of PTC cells (Figure 2A–D). Furthermore, we constructed xenograft mouse model to investigate the impact of OTUB1 suppression on PTC growth. As a result, our data revealed that stable OTUB1 interference in KTC‐1 triggered the decreased volume and weight of tumour (Figure 2E,F). These results implied that OTUB1 inhibition can suppress PTC cell growth.

### 3.3 Ectopic expression of OTUB1 facilitates PTC cell proliferation *in vitro* and *in vivo*

**FIGURE 3:**
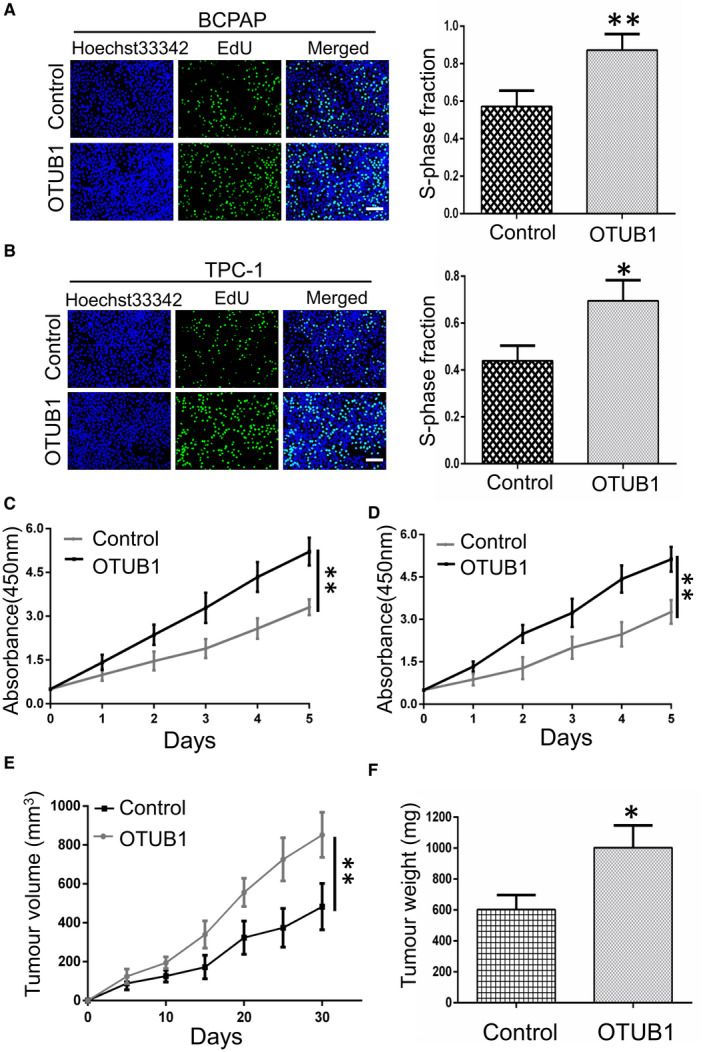
Ectopic expression of OTUB1 fosters PTC cell proliferation *in vitro* and *in vivo*. (A and B) EdU assay examining the proliferation capability for BCPAP and TPC‐1 cells transfected with the control or OTUB1 overexpression plasmids. (C and D) CCK‐8 assay was used to detect the growth ability of BCPAP and TPC‐1 cells with OTUB1 upregulation or not. (E and F) Tumour sizes and tumour weights of the control group or OTUB1‐overexpressing group of nude mice were measured and corresponding tumour growth curves were obtained. Data were mean ± SD of three independent determinations. **p < 0.01, ***p < 0.001 by *t* tests, scale bar, 100 μm

To further verify the function of OTUB1 in fostering PTC cells proliferation, gain‐of‐function assay of OTUB1 was conducted by using lentiviral vectors with overexpressing plasmids. We employed EdU proliferation assay and CCK‐8 assay to examine the growth ability of K1 and KTC‐1 cells with OTUB1 overexpression in comparison with the control group. Our results showed that OTUB1 upregulation significantly enhanced the proliferation ability of PTC cells (Figure 3A–D). Consistently, we constructed xenograft mouse model to examine the impact of OTUB1 upregulation on PTC proliferation. Our results demonstrated that OTUB1 overexpression in BCPAP triggered increased volume and weight of tumour (Figure 3E,F). Taken together, our results reveal the crucial role of OTUB1 upregulation in driving PTC cell proliferation.

### 3.4 The expression of OTUB1 is highly linked with EYA1 in PTC

**FIGURE 4:**
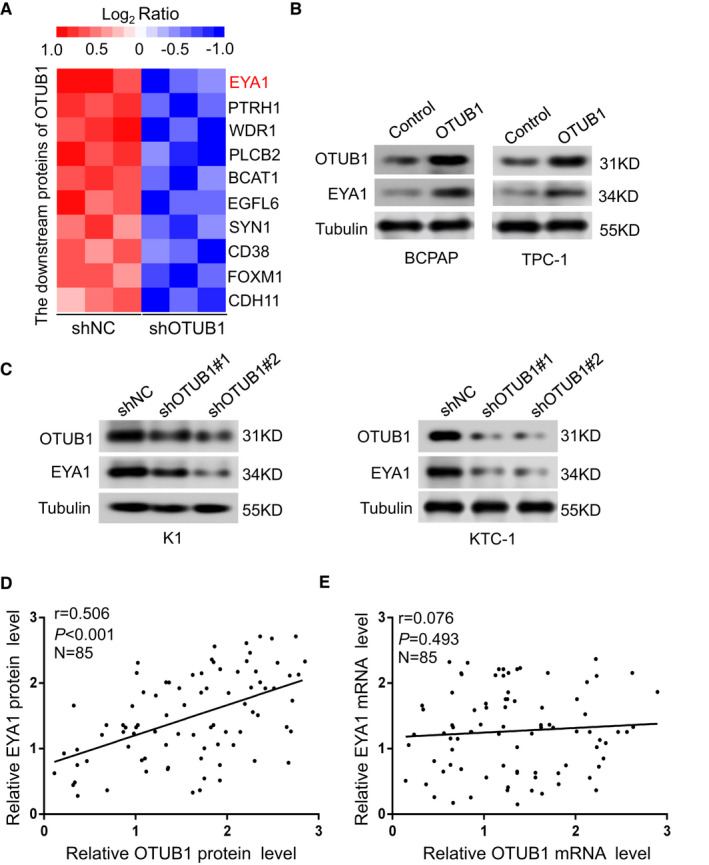
EYA1 protein level is dramatically related to OTUB1 expression in PTC. (A) Mass spectroscopic analysis listed the top 10 downregulated proteins with OTUB1 downregulation in KTC‐1 cells. (B) Western blot examining the expression of OTUB1 and EYA1 protein in BCPAP and TPC‐1 cells transfected with OTUB1‐overexpressing plasmids or the negative control. (C) Western blot showing the protein level of OTUB1 and EYA1 in K1 and KTC‐1 cells transfected with OTUB1 shRNA in comparison with the control group. (D) Scatter plots indicated no significant relationship between OTUB1 and EYA1 at the mRNA level in 85 PTC specimens. (E) Scatter plots demonstrated the positive correlation between OTUB1 and EYA1 at the protein level in 85 PTC samples

To further explore the downstream target of OTUB1 in PTC progression, we performed tandem mass tag (TMT)‐mass spectrometry proteomics analysis to examine the proteins expression patterns affected by OTUB1 knockdown in KTC‐1 cells. The data showed the decreased expression levels of multiple related proteins. Among the top ten downregulated proteins in heat map, EYA1 was selected as the most related protein of OTUB1 (Figure 4A). More importantly, EYA1 has been reported to drive thyroid carcinoma progression.^19^ To further confirm the correlation between OTUB1 and EYA1, we detected the protein level of EYA1 with OTUB1 overexpression or knockdown. Interestingly, our results showed that OTUB1 upregulation increased EYA1 protein level whereas knockdown of OTUB1 had the opposite effect (Figure 4B,C). To extend our investigations in human PTC specimens, the expressions of OTUB1 and EYA1 in PTC tissues were investigated. Our results revealed the significant correlation between OTUB1 and EYA1 protein levels (Figure 4D). In comparison, there was no correlation between OTUB1 and EYA1 mRNA levels (Figure 4E). These results suggest that OTUB1 expression is positively correlated with EYA1 protein level in PTC.

### 3.5 OTUB1 blocks EYA1 ubiquitination and stabilizes EYA1

**FIGURE 5:**
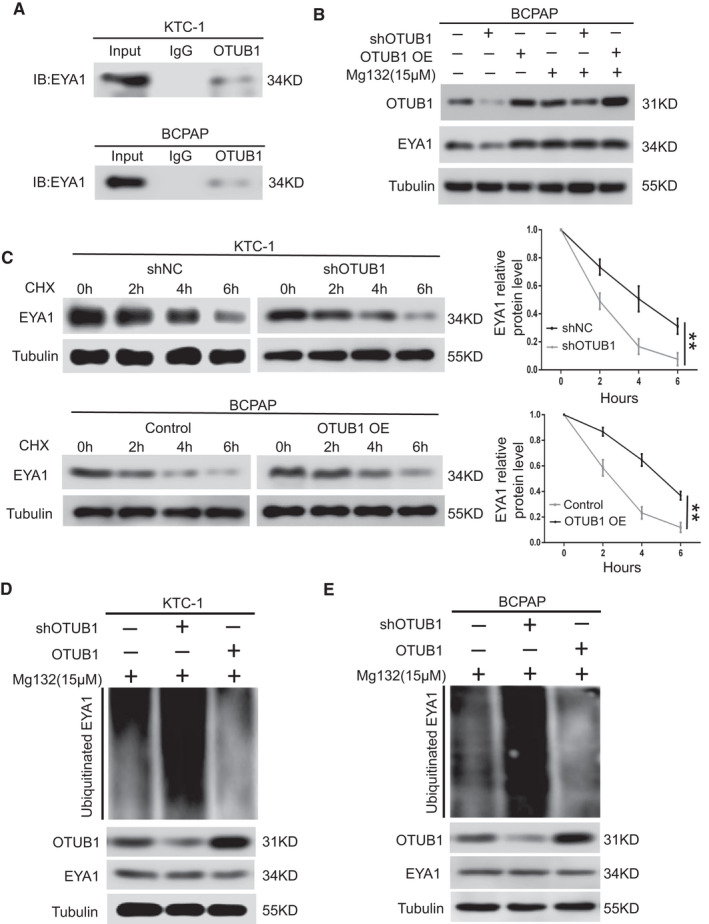
OTUB1 interacts with EYA1 and blocks EYA1 degradation. (A) co‐IP experiments indicating the direct interaction between endogenous OTUB1 and EYA1 in KTC‐1 and BCPAP cells. (B) BCPAP cells transfected with OTUB1 shRNA or OTUB1‐overexpressing plasmids were treated with MG132 (15 μM) or not. (C) KTC‐1 cells transfected with OTUB1 shRNA or shNC together with BCPAP cells stably overexpressing OTUB1 or the negative control were treated with 20 μM cycloheximide (CHX). Cells were collected at different time points, and EYA1 protein expression was detected. Right panel is quantification of the results of Western blot. (D and E) the downregulation or ectopic expression of OTUB1 altered the ubiquitination level of EYA1 in KTC‐1 and BCPAP cells. The cells in each group were treated with MG132 (15 μM). **p < 0.01 by *t* tests

To determine how OTUB1 interacts with EYA1 and affects protein expression of EYA1 in PTC cells, we carried out co‐IP experiments and deubiquitylation assay. EYA1 can be ubiquitinated and degraded through the ubiquitin‐proteasome pathway in mammalian cells.^20^ Given the role of OTUB1 in deubiquitylation, we speculated that OTUB1 might block the ubiquitination of EYA1 in PTC cells. Through co‐IP experiments in KTC‐1 and BCPAP cells, we found that OTUB1 could bind EYA1 directly (Figure 5A). Consistently, we employed proteasome inhibitor MG132 and translation inhibitor cycloheximide (CHX) to investigate whether OTUB1 is involved in the process of EYA1 degradation. Our results showed that OTUB1 had effective regulation on EYA1 protein level in the BCPAP cell not treated with MG132 while in the BCPAP cell treated with MG132, neither downregulation nor overexpression of OTUB1 had significant effect on protein level of EYA1 (Figure 5B). Furthermore, the degradation dynamics assay revealed that OTUB1 knockdown increased the degradation rate of EYA1 compared with the control group whereas OTUB1 overexpression showed the opposite effect (Figure 5C). At last, deubiquitylation assay showed that OTUB1 downregulation significantly increased the ubiquitination level of EYA1. In comparison, OTUB1 overexpression dramatically blocked the ubiquitination of EYA1 (Figure 5D,E). Collectively, these results indicate that OTUB1 can block the ubiquitination level of EYA1 effectively.

### 3.6 EYA1 is indispensable for OTUB1‐mediated PTC growth

**FIGURE 6:**
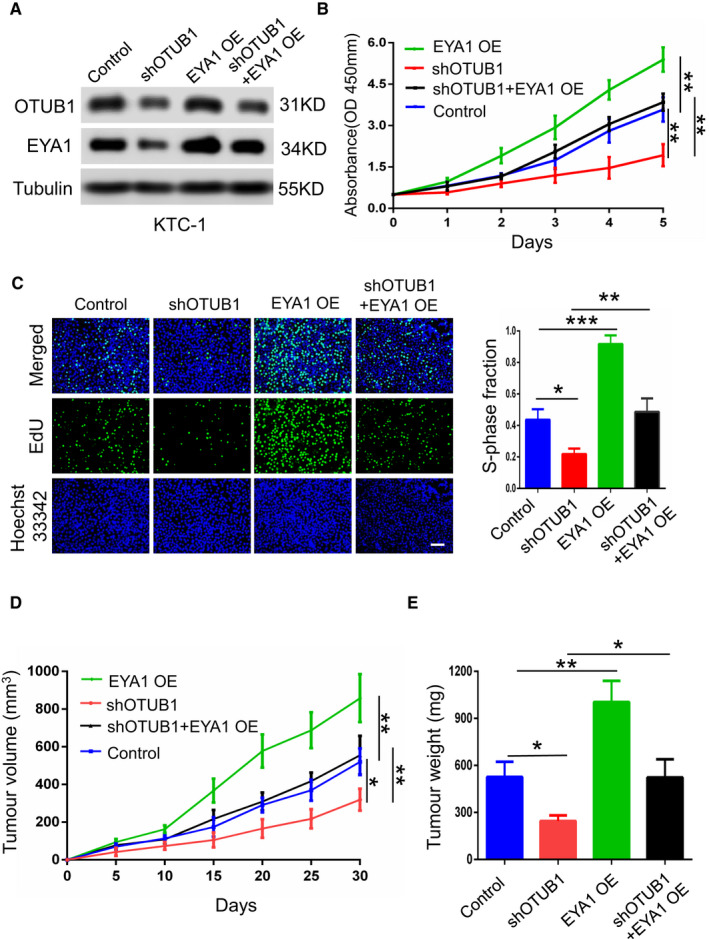
EYA1 is indispensable for PTC cells growth in OTUB1‐dependent manner. (A) Western blot shows that OTUB1 knockdown inhibited upregulated EYA1 expression in KTC‐1 cells. (B and C), CCK‐8 and EdU assay showing that OTUB1 knockdown abated KTC‐1 cell proliferation enhanced by EYA1 overexpression. (D and E) Tumour sizes and tumour weights of 20 nude mice (5 mice per group) were obtained, and correlated tumour growth curves were made in the rescue experiments. **p < 0.01, ***p < 0.001

To explore the underlying function of EYA1 in OTUB1‐derived PTC cells proliferation, we carried out rescue experiments and explored whether EYA1 was an essential downstream target of OTUB1 in PTC cells. In KTC‐1 cells, we transfected OTUB1 shRNA and EYA1 overexpression plasmids simultaneously. Interestingly, our results demonstrated that ectopic expression of EYA1 upregulated EYA1 protein level whereas OTUB1 knockdown abated this trend (Figure 6A). Then, we performed the rescue experiments through CCK‐8 assay and EdU assay. We found that EYA1 upregulation significantly enhanced the growth capability of KTC‐1 cells whereas OTUB1 downregulation suppressed this trend (Figure 6B,C). To further determine the biological function of OTUB1 and EYA1 *in vivo*, we set up tumorigenicity mouse models through subcutaneously injecting KTC‐1 cells with OTUB1 stably knockdown and/or EYA1 overexpression. Intriguingly, we observed that EYA1 overexpression dramatically facilitated tumour growth, but OTUB1 downregulation significantly suppressed this trend (Figure 6D,E). Herein, our results suggest that EYA1 is indispensable for PTC cell proliferation in OTUB1‐dependent manner.

## 4 DISCUSSION

For the first time, the current study identifies the crucial role of deubiquitinase OTUB1 in PTC and the molecular mechanism in regulating PTC growth. In this research, we demonstrate the following: (1) OTUB1 is aberrantly overexpressed in PTC tissues and cell lines; (2) OTUB1 significantly regulates PTC cells proliferation *in vitro* and *in vivo*; (3) OTUB1 expression is markedly correlated with EYA1 protein level in PTC cells and tissues; (4) OTUB1 can deubiquitinate EYA1 and inhibit EYA1 degradation; and (5) EYA1 plays an essential role in OTUB1‐mediated PTC cells growth. These findings reveal a novel role and underlying mechanism of OTUB1 in PTC proliferation and highlight the potential possibility of OTUB1 to become a therapeutic target.

Deubiquitinating enzymes (DUBs) are shown to be essential regulators of many cell‐signalling cascades in multiple cancers. Many DUBs can regulate the stability of key oncogenic proteins such as c‐Myc and Nrf2.^21^ Thus, it is critical to understand the molecular mechanisms about how DUBs regulate cancer progression. In recent years, increasing evidences have shown the oncogenic role of OTUB1, a member of the OTU subfamily of deubiquitinases, in multiple cancers. Previous studies demonstrated that OTUB1 is overexpressed in several carcinomas, including breast cancer,^22^ oesophageal cancer^23^ and ovarian cancer,^24^ but the function of OTUB1 in PTC remains elusive. Here, we demonstrated that OTUB1 was aberrantly overexpressed in PTC specimens and cell lines. To further investigate the function of OTUB1 in PTC, we performed a series of gain‐ and loss‐of‐function assays to identify the potential role of OTUB1 in fostering PTC growth. Our results indicated that OTUB1 downregulation inhibited PTC cells growth whereas OTUB1 overexpression showed the opposite trend. In keeping with our studies, Baietti et al. showed that OTUB1 overexpression drives lung adenocarcinoma cell growth.^25^ Additionally, it was reported that OTUB1 knockdown inhibits prostate cancer progression whereas OTUB1 upregulation has the opposite effect.^13^

OTUB1 appears to be a key regulator in some cancers. One study showed that OTUB1 is a crucial regulator of snail in oesophageal squamous cell carcinoma.^26^ Another study also suggested that OTUB1 mediates Forkhead Box M1 (FOXM1) in liver cancer cells.^27^ However, the downstream target of OTUB1 in PTC cells has not been reported. In our study, we identified the eyes absent homologue 1 (EYA1) protein as a potential downstream target of OTUB1 through mass spectrometry analysis. EYA1 was initially identified as a developmentally essential gene in drosophila, and loss of EYA1 contributes to absence or malformation of eyes.^28^ Additionally, EYA1 plays an essential role in DNA repair.^29^ Aberrant expression of EYA1 was found in multiple cancers, and it showed that EYA1 plays an oncogenic role in regulating progression of cancer.^30^, ^31^, ^32^ More importantly, increased protein level of EYA1 has been reported to enhance PTC cell proliferation.^19^ Thus, we investigated the correlation between EYA1 and OTUB1 in PTC cells and tissues. Our data indicated the significant correlation between OTUB1 and EYA1 in PTC cells. Interestingly, positive correlation between OTUB1 and EYA1 was determined in PTC tissues whereas no significant relationship was found at mRNA level. Additionally, it was reported that EYA1 can be stabilized at the protein level.^19^ Given the role of OTUB1 as a DUB, we speculated that EYA1 might be deubiquitinated by OTUB1.

Intriguingly, we found that OTUB1 can interact with EYA1 directly in KTC‐1 and BCPAP cells. Furthermore, one research has shown that EYA1 can be ubiquitinated and degraded through the ubiquitin‐proteasome pathway in mammalian cells.^20^ In this study, we further demonstrated that OTUB1 expression has impact on ubiquitin‐proteasome pathway of EYA1. Moreover, OTUB1 downregulation increased protein degradation rate and ubiquitination level of EYA1 whereas OTUB1 overexpression showed the opposite effect. In prostate cancer, OTUB1 can block the ubiquitination of cyclin E1 and keep it stable.^13^ Similarly, OTUB1 positively regulates PD‐L1 stabilization through deubiquitylation in human breast cancer.^12^ Our study indicated the novel role of OTUB1 in regulating EYA1 ubiquitination in PTC.

On the other hand, our findings also suggested that OTUB1 promoted PTC growth in an EYA1‐dependent manner. It also reported that EYA1 is essential in sine oculis homeobox 1 (SIX1)‐mediated PTC cells growth.^19^ Here, our results determined the critical role of EYA1 in OTUB1‐derived PTC cells growth. In our study, OTUB1 knockdown suppressed the protein level of EYA1 and PTC cells proliferation ability whereas ectopic expression of EYA1 significantly abated this trend. Additionally, our *in vivo* experiments also demonstrated that EYA1 was essential for OTUB1‐mediated PTC growth.

In conclusion, we have identified OTUB1 as a crucial enhancer in the process of PTC progression. The expression of OTUB1 is positively correlated with EYA1 protein level in PTC. OTUB1 functions as a deubiquitinase to block the degradation of EYA1 through ubiquitin‐proteasome pathway. In this respect, the stabilization of EYA1 protein contributes to enhanced PTC growth. Moreover, EYA1 is indispensable for OTUB1‐mediated PTC proliferation. OTUB1‐EYA1 axis may become a therapeutic target for PTC proliferation, and designing inhibitors targeting OTUB1‐EYA1 axis might be a promising approach to control PTC growth.

## CONFLICT OF INTEREST

The authors confirm that there are no conflicts of interest.

## AUTHOR CONTRIBUTIONS

**Peiyi Xie:** Investigation (lead); Software (equal); Writing‐original draft (lead). **Qing Chao:** Formal analysis (equal); Investigation (equal); Methodology (equal). **Jiuang Mao:** Investigation (equal); Software (equal). **Yue Liu:** Formal analysis (equal); Investigation (equal); Methodology (equal). **Jiayu Fang:** Methodology (equal). **Jing Xie:** Investigation (equal). **Jing Zhen:** Formal analysis (equal). **Yongqi Ding:** Investigation (equal). **Bidong Fu:** Methodology (equal). **Yun Ke:** Formal analysis (equal); Investigation (equal). **Da Huang:** Funding acquisition (lead); Project administration (equal); Resources (lead); Supervision (lead); Visualization (equal); Writing‐review & editing (lead).

## References

[1] Cabanillas ME , McFadden DG , Durante C . Thyroid cancer. Lancet. 2016;388(10061):2783‐2795. 27240885 10.1016/S0140-6736(16)30172-6

[2] Jankovic B , Le KT , Hershman JM . Hashimoto's thyroiditis and papillary thyroid carcinoma: is there a correlation? J Clin Endocrinol Metab. 2013;98(2):474‐482. 23293329 10.1210/jc.2012-2978

[3] Chou CK , Chen RF , Chou FF , et al. miR‐146b is highly expressed in adult papillary thyroid carcinomas with high risk features including extrathyroidal invasion and the BRAFV600E mutation. Thyroid. 2010;20(5):489‐494. 20406109 10.1089/thy.2009.0027

[4] Brown RL , de Souza JA , Cohen EEW . Thyroid cancer: burden of illness and management of disease. Journal of Cancer. 2011;2:193. 21509149 10.7150/jca.2.193 PMC3079916

[5] Davies L , Welch HG . Increasing incidence of thyroid cancer in the United States, 1973–2002. JAMA. 2006;295(18):2164‐2167. 16684987 10.1001/jama.295.18.2164

[6] Komander D , Clague MJ , Urbé S . Breaking the chains: structure and function of the deubiquitinases. Nat Rev Mol Cell Biol. 2009;10(8):550‐563. 19626045 10.1038/nrm2731

[7] Mevissen TET , Komander D . Mechanisms of deubiquitinase specificity and regulation. Annu Rev Biochem. 2017;86:159‐192. 28498721 10.1146/annurev-biochem-061516-044916

[8] Mevissen T , Hospenthal M , Geurink P , et al. OTU deubiquitinases reveal mechanisms of linkage specificity and enable ubiquitin chain restriction analysis. Cell. 2013;154(1):169‐184. 23827681 10.1016/j.cell.2013.05.046 PMC3705208

[9] Komander D , Clague MJ , Urbe S . Breaking the chains: structure and function of the deubiquitinases. Nat Rev Mol Cell Biol. 2009;10:550‐563. 19626045 10.1038/nrm2731

[10] He M , Zhou Z , Wu G , Chen Q , Wan Y . Emerging role of DUBs in tumor metastasis and apoptosis: Therapeutic implication. Pharm Ther. 2017;177:96‐107. 10.1016/j.pharmthera.2017.03.001 PMC5565705 28279784

[11] Zhou K , Mai H , Zheng S , et al. OTUB1‐mediated deubiquitination of FOXM1 up‐regulates ECT‐2 to promote tumor progression in renal cell carcinoma. Cell Biosci. 2020;10:50. 32257108 10.1186/s13578-020-00408-0 PMC7106863

[12] Zhu D , Xu R , Huang X , et al. Deubiquitinating enzyme OTUB1 promotes cancer cell immunosuppression via preventing ER‐associated degradation of immune checkpoint protein PD‐L1. Cell Death Differ. 2021;28:1773‐1789. doi:10.1038/s41418-020-00700-z 33328570 PMC8184985

[13] Zhang HH , Li C , Ren JW , et al. OTUB1 facilitates bladder cancer progression by stabilizing ATF6 in response to ER. Cancer Sci. 2021;112:2199‐2209. doi:10.1111/cas.14876 33686769 PMC8177800

[14] Liao Y , Wu N , Wang K , et al. OTUB1 promotes progression and proliferation of prostate cancer via deubiquitinating and stabling cyclin E1. Front Cell Dev Biol. 2021;8:617758. 33537306 10.3389/fcell.2020.617758 PMC7848094

[15] Ni Q , Chen J , Li X , et al. Expression of OTUB1 in hepatocellular carcinoma and its effects on HCC cell migration and invasion. Acta Biochim Biophys Sin (Shanghai). 2017;49(8):680‐688. 28575188 10.1093/abbs/gmx056

[16] Xie P , Wang H , Fang J , et al. CSN5 promotes carcinogenesis of thyroid carcinoma cells through ANGPTL2. Endocrinology. 2021;162(3):bqaa206. 33508120 10.1210/endocr/bqaa206

[17] Hu G , Yan C , Xie P , Cao Y , Shao J , Ge J . PRMT2 accelerates tumorigenesis of hepatocellular carcinoma by activating Bcl2 via histone H3R8 methylation. Exp Cell Res. 2020;394(2):112152. 32574605 10.1016/j.yexcr.2020.112152

[18] Xie P , Wang H , Xie J , et al. USP7 promotes proliferation of papillary thyroid carcinoma cells through TBX3‐mediated p57KIP2 repression. Mol Cell Endocrinol. 2020;518:111037. 32966862 10.1016/j.mce.2020.111037

[19] Kong D , Li A , Liu Y , et al. SIX1 activates STAT3 signaling to promote the proliferation of thyroid carcinoma via EYA1. Front Oncol. 2019;9:1450. 31921695 10.3389/fonc.2019.01450 PMC6933607

[20] Musharraf A , Kruspe D , Tomasch J , Besenbeck B , Englert C , Landgraf K . BOR‐syndrome‐associated Eya1 mutations lead to enhanced proteasomal degradation of Eya1 protein. PLoS One. 2014;9(1):e87407. 24489909 10.1371/journal.pone.0087407 PMC3906160

[21] Zhang J , Ren P , Xu DA , et al. Human UTP14a promotes colorectal cancer progression by forming a positive regulation loop with c‐Myc. Cancer Lett. 2019;441:106‐115. 10.1016/j.canlet.2018.10.010 30343112

[22] Karunarathna U , Kongsema M , Zona S , et al. OTUB1 inhibits the ubiquitination and degradation of FOXM1 in breast cancer and epirubicin resistance. Oncogene. 2016;35(11):1433‐1444. 26148240 10.1038/onc.2015.208 PMC4606987

[23] Sun J , Deng Y , Shi J , Yang W . MicroRNA‐542‐3p represses OTUB1 expression to inhibit migration and invasion of esophageal cancer cells. Mol Med Rep. 2020;21(1):35‐42. 31939620 10.3892/mmr.2019.10836 PMC6896300

[24] Wang S , Ning Y , Wei P , et al. The non‐coding RNA OTUB1‐isoform2 promotes ovarian tumour progression and predicts poor prognosis. J Cell Mol Med. 2018;22(10):4794‐4806. 30044532 10.1111/jcmm.13733 PMC6156285

[25] Baietti MF , Simicek M , Abbasi Asbagh L , et al. OTUB1 triggers lung cancer development by inhibiting RAS monoubiquitination. EMBO Mol Med. 2016;8(3):288‐303. 26881969 10.15252/emmm.201505972 PMC4772950

[26] Zhou H , Liu Y , Zhu R , et al. OTUB1 promotes esophageal squamous cell carcinoma metastasis through modulating Snail stability. Oncogene. 2018;37(25):3356‐3368. 29559747 10.1038/s41388-018-0224-1

[27] Higurashi M , Maruyama T , Nogami Y , et al. High expression of FOXM1 critical for sustaining cell proliferation in mitochondrial DNA‐less liver cancer cells. Exp Cell Res. 2020;389(1):111889. 32032602 10.1016/j.yexcr.2020.111889

[28] Bonini NM , Bui QT , Gray‐Board GL , Warrick JM . The Drosophila eyes absent gene directs ectopic eye formation in a pathway conserved between flies and vertebrates. Development. 1997;124(23):4819‐4826. 9428418 10.1242/dev.124.23.4819

[29] Farrell AW , Halliday GM , Lyons JG . Chromatin structure following UV‐induced DNA damage‐repair or death? Int J Mol Sci. 2011;12(11):8063‐8085. 22174650 10.3390/ijms12118063 PMC3233456

[30] Cai S , Cheng X , Liu YI , et al. EYA1 promotes tumor angiogenesis by activating the PI3K pathway in colorectal cancer. Exp Cell Res. 2018;367(1):37‐46. 29496520 10.1016/j.yexcr.2018.02.028

[31] Eisner A , Pazyra‐Murphy M , Durresi E , et al. The Eya1 phosphatase promotes Shh signaling during hindbrain development and oncogenesis. Dev Cell. 2015;33(1):22‐35. 25816987 10.1016/j.devcel.2015.01.033 PMC4418443

[32] Nikpour P , Emadi‐Baygi M , Emadi‐Andani E , Rahmati S . EYA1 expression in gastric carcinoma and its association with clinicopathological characteristics: a pilot study. Med Oncol. 2014;31(5):955. 24729159 10.1007/s12032-014-0955-y

